# “Almost Bleeding to Death”: The Conundrum of Acquired Amegakaryocytic Thrombocytopenia

**DOI:** 10.1155/2014/806541

**Published:** 2014-02-06

**Authors:** Gabrielle Elena Brown, Hani M. Babiker, Carlos L. Cantu, Andrew M. Yeager, Ravitharan Krishnadasan

**Affiliations:** ^1^University of Arizona College of Medicine, 1501 North Campbell Avenue, Tucson, AZ 85724, USA; ^2^Division of Hematology-Oncology, Department of Medicine, University of Arizona College of Medicine, 1501 North Campbell Avenue, Tucson, AZ 85724, USA; ^3^University of Arizona Cancer Center, 1515 North Campbell Avenue, Tucson, AZ 85724, USA; ^4^Department of Pathology, University of Arizona College of Medicine, 1501 North Campbell Avenue, Tucson, AZ 85724, USA; ^5^Department of Pediatrics, University of Arizona College of Medicine, 1501 North Campbell Avenue, Tucson, AZ 85724, USA

## Abstract

Acquired amegakaryocytic thrombocytopenia (AAT) is a rare hematological disorder causing severe thrombocytopenia and bleeding. Previous in vitro studies postulated both cell-mediated suppression of megakaryocytopoiesis in early megakaryocytic progenitor cells and humoral-mediated suppression by anti-thrombopoietin antibodies as possible etiologies of AAT. Patients with AAT usually present with severe bleeding and thrombocytopenia that is unresponsive to steroids and intravenous immunoglobulin (IVIG). Although standard guidelines have not been established for management of AAT, a few case reports have indicated a response to immunosuppressive treatment. The prompt recognition of this disease entity is essential in view of the substantial risk of morbidity and mortality from excessive bleeding. We report a case of AAT successfully treated with equine antithymocyte globulin (ATG) and cyclosporine (CSP).

## 1. Case Presentation

**Figure 1:**
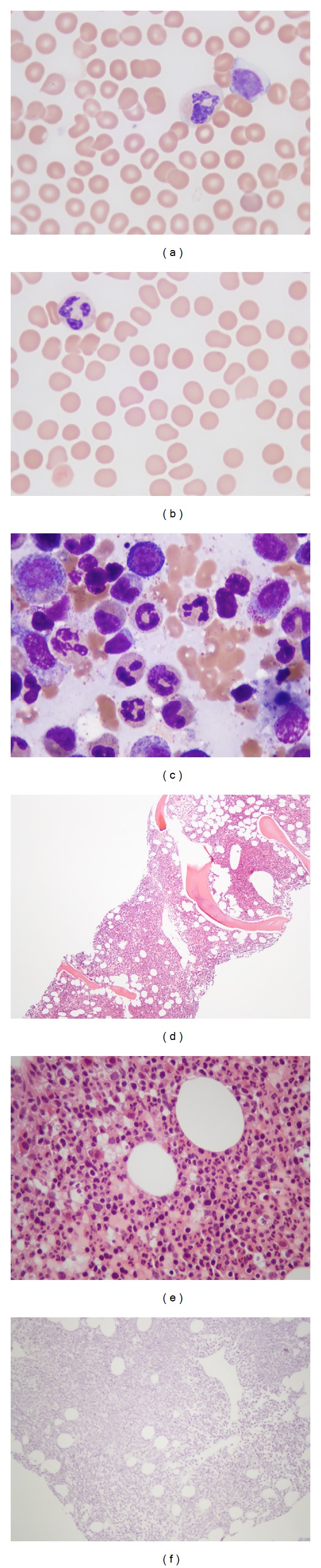
((a)-(b)) The peripheral blood shows normal mature neutrophils and lymphocytes with normal red blood cell morphology. Rare morphologically normal platelets are identified. (c) The bone marrow aspirate shows a spectrum of normal maturation in erythroid and granulocytic precursors. No abnormal cell population is identified. (d) The bone marrow core biopsy cellularity is appropriate for age. (e) The bone marrow interstitium does not show the presence of megakaryocytes. (f) Immunohistochemistry for CD61, a megakaryocytic antigen, does not show the presence of megakaryocytes on the core biopsy tissue section.

A 40-year-old woman with a past medical history of migraine headaches presented to her primary care physician with the chief complaint of “I am almost bleeding to death” and endorsed a history of fatigue, easy bruising, and frequent nosebleeds. Her only medication was an oral contraceptive. A complete blood count (CBC) revealed a platelet count of 12 × 10^9^/L (normal, 150–425 × 10^9^/L), and she was referred to a hematologist. She was initially diagnosed with idiopathic thrombocytopenia purpura (ITP) and was treated with prednisone 60 mg per day for one week, without improvement in platelet count. A bone marrow biopsy (BMB) revealed a hypercellular marrow (75%) with trilineage hematopoiesis but with decreased megakaryocytes; maturation of erythroid and myeloid elements were normal. Because of persistent thrombocytopenia, her treatment regimen was modified to include weekly platelet infusions and prednisone. After each platelet transfusion, there was an increment in platelet level from approximately 12 × 10^9^/L to approximately 70 × 10^9^/L, followed by a return to baseline level of approximately 12 × 10^9^/L over the following 2-3 days. The patient was then referred to our hematology service for further evaluation. On physical examination, she had scattered petechiae and ecchymosis on her upper and lower extremities, but no rashes, hepatosplenomegaly, or lymphadenopathy. A CBC showed a platelet count of 25 × 10^9^/L, and review of the peripheral blood smear showed rare giant platelet forms and the absence of platelet clumps. In addition, a leukocytosis was present with a white blood cell count of 13.9 × 10^9^/L (normal, 3.4–10.4 × 10^9^/L), with absolute neutrophil count (ANC) of 12.09 × 10^9^/L (normal, 1.8–7.7 × 10^9^/L), and with an absolute lymphocyte count (ALC) of 1.39 × 10^9^/L (normal, 1.0–4.8 × 10^9^/L), and other labs were normal. A repeat BMB at our institution showed a normocellular marrow, no evidence of myelodysplasia, and absence of megakaryocytes, confirmed by lack of immunohistochemical staining for CD61, consistent with a diagnosis of AAT (Figure 1). Cytogenetic analysis of the bone marrow revealed a normal female karyotype (46,XX). Fluorescent in situ hybridization analysis did not show evidence of deletion 5q31, monosomy 7, deletion 7q31, trisomy 8, or deletion 20q. A monoclonal T-cell population was detected by PCR.

**Figure 2:**
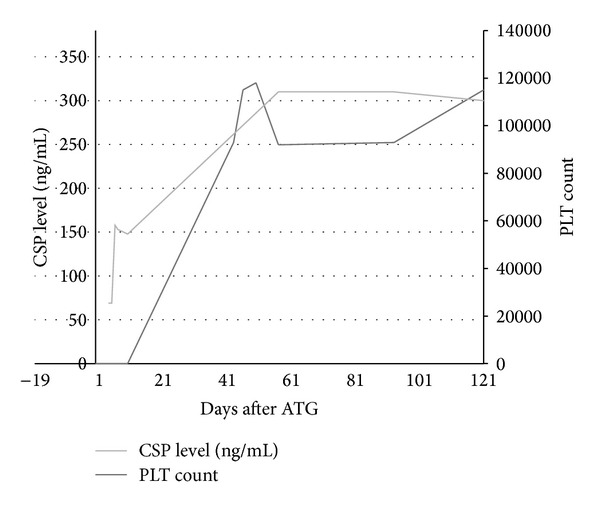
Patient's platelet counts over 121 days following treatment with ATG and CSP. Displayed in dark and light grey lines are platelets count and CSP levels, respectively.

The patient was admitted and received a four-day course of equine ATG (40 mg/kg/day) followed by a 6-month outpatient course of CSP. She also received a two-week course of methylprednisolone to ameliorate symptoms of serum sickness from ATG administration. She was discharged on a tapering steroid course and CSP 350 mg orally twice a day with a target trough level of 200–250 ng/mL. The patient required one platelet transfusion shortly after discharge. By 4 months after initiation of ATG/CSP treatment the platelet level had increased to 115, 000 × 10^9^/L and remained stable thereafter (Figure 2).

## 2. Discussion

The differential diagnosis for acquired thrombocytopenia is broad and includes splenic sequestration, decreased production from viral infections, chemotherapy, toxins, irradiation, aplastic anemia, myelofibrosis, paroxysmal nocturnal hemoglobinuria, or leukemias. Other etiologies of thrombocytopenia relate to increased destruction of platelets, as in ITP, TTP, autoimmune diseases, hemolytic uremic syndrome, medications, severe bacterial infections, and pregnancy. Our patient had no splenomegaly, exposure to toxic chemicals, symptoms of infection, or blasts in the peripheral smear, and her pregnancy test was negative. She had an isolated finding of thrombocytopenia, for which our differential diagnosis given her physical exam and diagnostic results included ITP, TTP, myelodysplastic syndrome (MDS), drug-induced thrombocytopenia, congenital thrombocytopenia, and AAT.

ITP, characterized by a decreased platelet count and mucocutaneous bleeding, is one of the most common causes of thrombocytopenia and diagnosis is established clinically via exclusion of other etiologies [1]. There is a female predominance in patients under the age of 65, after which there is no significant difference in gender-related incidence [1]. The primary mechanism involves antiplatelet antibody associated platelet clearance, although alternate or supplementary mechanisms of immune dysregulation, altered platelet production, and genetic predisposition have been characterized [1, 2]. Although the patient's age and gender initially raised suspicion for ITP, the poor response to treatment with systemic steroids prompted further consideration of other etiologies. The absence of megakaryocytes on BMB was also inconsistent with a diagnosis of ITP. Bone marrow biopsies obtained from patients with ITP usually show expansion of the megakaryocytic compartment with a spectrum of maturing megakaryocytes.

Thrombotic thrombocytopenic purpura (TTP) is an acquired thrombotic microangiopathy characterized by a pentad of renal and neurologic symptoms, thrombocytopenia, fever, and microangiopathic hemolytic anemia [3, 4]. Acquired TTP is associated with decreased metalloprotease ADAMTS-13 activity, which is a protease responsible for cleaving von Willebrand factor (vWF) multimers; failure to degrade vWF results in platelet aggregation and subsequent thrombocytopenia [3]. This diagnosis was considerably lower on the differential list as the patient lacked clinical signs of fever, neurologic abnormalities, or renal dysfunction, and the peripheral smear lacked schistocytes. In addition, as seen with ITP, patients with TTP tend to also show an expansion of the megakaryocytic compartment that was not appreciated on repeat BMB.

Neoplastic, iatrogenic, and congenital causes of thrombocytopenia were also considered, including MDS, drug-induced thrombocytopenia, and inherited thrombocytopenia. MDS was effectively ruled out with the negative MDS cytogenetic panel results and the lack of dysplasia of all cell lineages. In addition, MDS usually presents in patients with a median age of 60 to 75 years and rarely in patients younger than 50. Many medications have been implicated in drug-induced thrombocytopenia, and thrombocytopenia often occurs in the first 1-2 weeks after initiating the medication [5]. A limited number of case reports have highlighted an association between an implantable contraceptive device and TTP; however, no published cases have implicated oral contraceptives as a cause of thrombocytopenia [6]. There was no temporal relationship between the patient's long-standing use of oral contraceptives and the current presentation. A congenital cause is less likely in this patient given her negative family history, normal preceding platelet counts, age at presentation, and the indolent course of her illness.

AAT is a rare disorder that is characterized by severe thrombocytopenia and diminished or absent megakaryopoiesis in the setting of otherwise normal bone marrow [7]. Patients usually present with bleeding and thrombocytopenia not responding to treatment with steroids or IVIG. Patients can achieve a durable remission, develop a long relapsing and remitting course, progress to aplastic anemia, or require a bone marrow transplantation to achieve remission [8–10]. AAT may be associated with other hematologic and rheumatologic conditions, including MDS, aplastic anemia, acute myeloid leukemia, and systemic lupus erythematosus [11, 12]. The exact mechanism has not been elucidated; however, the presence of antithrombopoietin IgG antibodies in patients with AAT suggests a dysregulated humoral immunity [13]. Dysfunction of cell-mediated immunity has also been postulated, in which monoclonal T-lymphocytes obtained from a patient with AAT were found to inhibit megakaryocyte lineage in vitro, but not other cell lineages [14]. Our patient also had a monoclonal T-cell population detected on PCR, and the isolated absence of megakaryocytes in bone marrow is consistent with AAT. Although cytogenetic abnormalities had been associated with AAT, their precise role in pathogenesis and prognosis is unknown. Prior case reports of AAT reported positivity of Ph and 5q-chromosomes, with the latter case progressing to acute myelomonocytic leukemia [15, 16].

## 3. Management

Standard treatment guidelines have not been established for AAT, and few case reports have been published regarding the management of this disorder. Based on the proposed humoral immunity mechanism, various immunosuppressive treatment approaches have been utilized in patients with AAT.

Several case series have reported successful treatment with CSP as a monotherapy or in combination with other immunosuppressive agents [7, 17–19]. One case series documented the clinical course of 4 patients with AAT who were treated with ATG at 40 mg/kg/day for four days and then with an extended course of CSP [7]. Three of the four patients achieved a response within 6 weeks and remained in complete remission at 60 months after-treatment [7]. A similar study evaluated a trial of combination of ATG and CSP in patients with severe AAT/aplastic anemia, although a high incidence of relapse was observed [19].

Other therapies for AAT have included rituximab [11], danazol [20], azathioprine [12], and bone marrow transplant [10], with variable success. Treatment with rituximab resulted in a rapid platelet response in one case report, although the patient ultimately relapsed [11]. Danazol was used in a case of cyclic AAT, with complete remission achieved for at least 10 months [20]. Azathioprine treatment resulted in resolution of clinical symptoms at 4 weeks and complete remission at 6 weeks [12]. A more aggressive approach with myeloablative chemotherapy followed by a fully HLA-matched allogeneic bone marrow transplant has also been reported to be successful [10].

This case report highlights the presentation, potential pathogenetic mechanisms, and diagnosis of AAT. Additionally, our observations confirm the treatment of this rare disease entity with combined immunosuppression consisting of ATG and CSP.
